# An immunoresponsive three-dimensional urine-tolerant human urothelial model to study urinary tract infection

**DOI:** 10.3389/fcimb.2023.1128132

**Published:** 2023-03-27

**Authors:** Nazila V. Jafari, Jennifer L. Rohn

**Affiliations:** Department of Renal Medicine, Division of Medicine, University College London, London, United Kingdom

**Keywords:** 3D human infection models, urothelium, urinary tract infection, CD markers, uroplakins, cytokeratins, tight junctions, innate immunity

## Abstract

**Introduction:**

Murine models of urinary tract infection (UTI) have improved our understanding of host-pathogen interactions. However, given differences between rodent and human bladders which may modulate host and bacterial response, including certain biomarkers, urothelial thickness and the concentration of urine, the development of new human-based models is important to complement mouse studies and to provide a more complete picture of UTI in patients.

**Methods:**

We originally developed a human urothelial three-dimensional (3D) model which was urine tolerant and demonstrated several urothelial biomarkers, but it only achieved human thickness in heterogenous, multi-layered zones and did not demonstrate the comprehensive differentiation status needed to achieve barrier function. We optimised this model by altering a variety of conditions and validated it with microscopy, flow cytometry, transepithelial electrical resistance and FITC-dextran permeability assays to confirm tissue architecture, barrier integrity and response to bacterial infection.

**Results:**

We achieved an improved 3D urine-tolerant human urothelial model (3D-UHU), which after 18-20 days of growth, stratified uniformly to 7-8 layers comprised of the three expected, distinct human cell types. The apical surface differentiated into large, CD227+ umbrella-like cells expressing uroplakin-1A, II, III, and cytokeratin 20, all of which are important terminal differentiation markers, and a glycosaminoglycan layer. Below this layer, several layers of intermediate cells were present, with a single underlying layer of CD271+ basal cells. The apical surface also expressed E-cadherin, ZO-1, claudin-1 and -3, and the model possessed good barrier function. Infection with both Gram-negative and Gram-positive bacterial classes elicited elevated levels of pro-inflammatory cytokines and chemokines characteristic of urinary tract infection in humans and caused a decrease in barrier function.

**Discussion:**

Taken together, 3D-UHU holds promise for studying host-pathogen interactions and host urothelial immune response.

## 1 Introduction

The urinary tract is the most common site of bacterial infection in humans, and urinary tract infection (UTI) affects ~150 million people around the world each year. Approximately 40% of women and 12% of men experience a symptomatic UTI during their lifetime, although infants and children are also susceptible. While acute UTIs are usually self-limiting, a quarter of affected women will suffer recurrent UTI despite treatment within 6-12 months (Nielubowicz and Mobley, 2010; Flores-Mireles et al., 2015).

The most apparent function of the urinary bladder is to store and void large volumes of urine. It is comprised of three tissue layers: the luminal urothelium, the lamina propria, and the detrusor muscle. The urothelium is a stratified, transitional epithelium that lines the surface of the renal pelvis, ureters, urinary bladder and proximal urethra. It serves as a barrier that prevents the diffusion of toxic substances such as acid and urea and defends against pathogens from the external environment (Winder et al., 2014; Osborn and Kurzrock, 2015; Balsara and Li, 2017; Jafari and Rohn, 2022). The urothelium consists of three cell types: the undifferentiated basal cells, intermediate cells and terminally differentiated luminal umbrella cells (Wu et al., 2009; Jaimes-Parra et al., 2016). Forming a single layer along the basement membrane, the basal cells are the smallest (5-10 μm in diameter) and are the most abundant cell population in adult urothelium (Keshtkar et al., 2007; Balsara and Li, 2017; Dalghi et al., 2020). The intermediate cells lie directly above the basal cells and are considerably larger (20 μm in diameter). In the urinary bladder of humans there are up to five intermediate cell layers, whereas in rodents only one layer is present (Winder et al., 2014; Yamany et al., 2014; Dalghi et al., 2020). Facing the luminal surface are large (50-120 μm in diameter), hexagonal and highly specialized cells known as superficial or umbrella cells (Balsara and Li, 2017; Jackson et al., 2020).

The apical surface of the umbrella cells is covered by a crystalline lattice comprising four key uroplakin proteins (UPK1A, UPK1B, UPKII, UPKIII) that together form a unique asymmetric unit membrane (AUM) plaque (Wu et al., 2009; Balsara and Li, 2017). Uroplakins are considered to be terminal differentiation markers of the urothelium. They initially form heterodimers (UPK1A-UPKII and UPK1B-UPKIII), then the resulting moieties interact to form heterotetradimers. Six heterotetradimers assemble a 16 nm protein particle arranged into a hexagonal lattice and are presented to the apical membrane which decorates up to 90% of the luminal surface (Jackson et al., 2020). In rodents, uroplakins are expressed within all urothelial layers, whilst in humans they are primarily expressed in the umbrella cells, highlighting species specificity in their expression pattens (Yu and Hill, 2011).

The urothelium also expresses several cytokeratins including cytokeratin 5 (CK5), CK7, CK8, CK13, CK14, and CK20. Cytokeratin expression varies in the urothelium depending on its location and some are expressed in a differentiation-specific manner, such as CK20 which is highly expressed in the umbrella cells (Moll et al., 1988; Southgate et al., 1999; Yamany et al., 2014; Dalghi et al., 2020). Despite the multilayer structure of the urothelium, the outermost umbrella cell layer forms the barrier to pathogens, urine and its associated metabolites, solutes, and water. This barrier itself is multifaceted, comprised of the apical membrane, the umbrella cell tight junction (containing proteins such as occludins and claudins), and the glycosaminoglycan (GAG) layer covering the umbrella cells (Khandelwal et al., 2009; Birder and Andersson, 2013).

The impermeable barrier of the umbrella cells together with protective glycan layer and frequent unidirectional flow of urine discourages bacterial adherence to the urothelium. Other factors such as changes in urine osmolarity, pH, soluble IgA, uromodulin (Tamm-Horsfall urinary glycoprotein), iron chelating siderophores and antimicrobial peptides (AMPs) can additionally limit bacterial attachment to the urothelium (Becknell et al., 2015). Furthermore, the urothelium employs another defence mechanism to reduce bacterial load by undergoing cell death and cell exfoliation into the urine. This allows the removal of cells that are associated with adherent and intracellular bacteria (Elliott et al., 1985; Cheng et al., 2016).

The urothelium also expresses multiple toll-like receptors (TLRs) which recognise pathogen-associated molecular patterns (PAMPs) (Akira et al., 2006; Zhang and Schluesener, 2006; Kawai and Akira, 2010). The activation of TLRs triggers the production of inflammatory mediators such as cytokine and chemokines. The common TLRs identified in the urinary tract include TLR-2, TLR-3, TLR-4, TLR-5, and TLR-9 (with TLR-11 only in mice) (Song and Abraham, 2008). Studies have reported that the normal human urothelium expresses TLR-5 weakly, TLR-2, TLR-3, and TLR-7 moderately, and TLR-4 and TLR-9 strongly (Ayari et al., 2011; Larue et al., 2013).

Although murine models of UTI have proven to be a powerful tool for advancing our understanding of UTI pathogenesis (Hung et al., 2009), mouse models have limitations (Murray et al., 2021). Physiological, structural, and genetic differences can limit how well mice reproduce key aspects of disease pathogenesis or a pathogen’s ability to replicate causing human-like diseases (Sarkar and Heise, 2019). Development of new treatments not only requires an in-depth understanding of disease pathogenesis but importantly, access to appropriate model systems to study host-pathogen interactions. While *in vitro* cell models are limited in terms of immune response and systemic crosstalk, their human microenvironmental context nevertheless can provide information complementary to animal studies (Ingber, 2020). Indeed, biomimetic *in vitro* models of the human urothelium have advanced significantly in recent years, offering vital information, but most are short-lived when in a urine environment (Andersen et al., 2012; Baker et al., 2014). This is a significant disadvantage, as urine is the natural context in which uropathogens interact at the host cell interface. We overcame this problem in our previous model (Horsley et al., 2018), but the urothelium was non-homogeneous in terms of differentiated surface coverage, and possibly as a result, the barrier function was not robust. Here, we report an improved terminally differentiated urine-tolerant human urothelium, 3D-UHU, that can stratify to a human urothelial thickness of 7-8 layers, exhibits the key biomarkers, possesses good barrier function and secretes appropriate human cytokines/chemokines in response to uropathogenic isolates.

## 2 Materials and methods

### 2.1 Human bladder epithelial progenitor cell culture

All models were grown with HBLAK human bladder progenitor cells (CELLnTEC, Switzerland) according to the online CELLnTEC protocol (https://cellntec.com/products/resources/protocols/culture/). HBLAK are spontaneously immortalised, non-transformed cells, originally derived from a male patient having benign prostatic hyperplasia surgery; if used within the correct passage window (see below), the cells behave like progenitor cells and can differentiate into a stratified urothelium. Briefly, the frozen commercial vial, which is cryo- preserved at approximately passage 25, was thawed and transferred to a cell culture vessel containing pre-equilibrated CnT-Prime medium (CnT-PR, CELLnTEC), incubated at 37°C and 5% CO_2_. HBLAK cells were passaged when they reached 70 to 90% confluency, detached using only Accutase and seeded at the recommended density (4000 cells cm^2^). During the course of cell culture, no serum, antibiotics and/or antifungals were used, and care was taken that cells never exceeded 90% confluency, as this can impair subsequent differentiation. Cells were propagated to passage 4 (otherwise known as passage 29, if including passages prior to receipt from the company), then frozen vials were prepared following the CELLnTEC freezing protocol.

### 2.2 Establishment of 3D-UHU

Human 3D bladder cultures were prepared as described previously (Hubbard et al., 2019), with some modifications. Briefly, HBLAK cells were used only within passage 8-12 after thawing the above-mentioned passage 4 stock and subsequent propagation (equivalent to passages 33-37 if including passages prior to vial receipt). Cells were seeded onto polycarbonate Transwell inserts (in a 12-well plate) with a pore size of 0.4 µm (VWR, United Kingdom) at the recommended seeding density (2 x 10^5^ cells per insert) (CELLnTEC, 3D Culture Protocol). 1.5 ml of CnT-PR medium was added to the basolateral chamber of the inserts and 0.5 ml to the apical chamber so that the medium levels were equal. Because the membranes are translucent and confluency cannot be confirmed by microscopy, we checked confluency by sacrificing a parallel well and staining with 4′,6-diamidino-2-phenylindole (DAPI) or equivalent to allow visualisation of cell confluence. Rare cultures that did not reach 100% confluence after 48 h were discarded, as our experience is that these will not terminally differentiate. Once cells were confluent, CnT-PR was replaced with 3D Barrier Medium (CnT-PR-3D, CELLnTEC) and incubated for 15-16 h so cells could form intercellular adhesion structures. 3D cultures were initiated by completely replacing apical CnT-PR-3D with filter-sterilized human urine (pooled gender) (BioIVT, UK) and fresh CnT-PR-3D medium in the basolateral chamber (tapering in the urine is not required), with the same volumes as before. Urine and CnT-PR-3D medium were changed at regular intervals (three times per week, or twice if ThinCert cell culture plates were used) and 3D cultures were fully stratified and differentiated on days 18-20. The cultures remained urine-tolerant throughout this period as assessed by their low shedding, healthy morphology and ultrastructure in subsequent experiments (see below results).

### 2.3 Characterisation of CD cell surface antigens

To profile 3D-UHU cluster of differentiation (CD) cell surface markers, terminally differentiated cultures were dissociated using Accutase solution (Merck, UK). Cells were washed with PBS and filtered (100 µm) to prepare single cell suspensions. BD Horizon Fixable Viability Stain 780 solution (1:1000) was added to the cell suspension and incubated at room temperature (RT) for 15 min. Cells were washed and blocked with blocking buffer plus Fc receptor blocking antibody (Clone Fc1.3216, BD Biosciences, UK). Cells were incubated at RT for 10 min then centrifuged (800 g, 3 min). BD Horizon Brilliant Stain Buffer was added to each sample followed by the following antibodies at the optimised volume per test (**Supplementary Table 1**): CD9 (BV421, Clone M-L13), CD44 (Alexa Fluor 700, Clone G44-26), CD47 (BV786, Clone B6H12), CD49b (Alexa Fluor 647, Clone AK-7), CD59 (BUV 395, Clone p282), CD63 (PE-Cy7, Clone H5C6), CD95 (BUV737, Clone DX2), CD104 (BV480, Clone 439-9B), CD227 (BV650, Clone HMPV), CD271 (BV711, Clone L128). Cells were incubated at 4°C in the dark for 30 min then washed and resuspended in wash buffer with 1% formaldehyde solution. Data acquisition was performed using Cytek Aurora equipped with 5 lasers. The flow cytometry results were analysed using FlowJo™ v10.8 Software (BD Life Sciences).

### 2.4 Immunostaining of 3D-UHU model and imaging

Prior to immunofluorescence staining, 3D-UHU cultures were fixed with 4% methanol-free formaldehyde (Thermo Fisher Scientific, UK) overnight at 4°C. Membranes were excised with a scalpel and placed in a 24-well plate and permeabilised in 0.2% Triton-X100 (Sigma-Aldrich, UK) in PBS for 20 min at RT. Membranes were washed with PBS then blocked with 5% normal goat serum (Thermo Fisher Scientific) at RT for 1 h. 3D cultures were incubated at 4°C overnight with primary antibodies in 1% bovine serum albumin (BSA)/PBS as follows: rabbit anti-uroplakin-1A (UPK1A) polyclonal antibody (1:100 dilution, PA5-49668, Thermo Fisher Scientific), rabbit anti-uroplakin-II (UPKII) polyclonal antibody (1:100 dilution, NBP2-38904, Novus biologicals); mouse anti-uroplakin-III (UPKIII) monoclonal antibody (1:50 dilution, sc-166808, Santa Cruz); mouse anti-cytokeratin 8 (CK8) monoclonal antibody (1:50 dilution, MA1-06317, Thermo Fisher Scientific); rabbit anti-cytokeratin 13 (CK13) polyclonal antibody (1 µg/ml, PA5-83165, Thermo Fisher Scientific); rabbit anti-cytokeratin 20 (CK20) polyclonal antibody (1:100 dilution, PA5-22125, Thermo Fisher Scientific); rabbit anti-E-cadherin polyclonal antibody (1:200 dilution, PA5-32178, Thermo Fisher Scientific); rabbit anti- zonula occludens-1 (ZO-1) polyclonal antibody (2.5 µg/ml, 40-2200, Thermo Fisher Scientific); mouse anti-claudin 1 monoclonal antibody (2 µg/ml, 37-4900, Thermo Fisher Scientific); rabbit anti-claudin 3 polyclonal antibody (1:100 dilution, PA5-16867, Thermo Fisher Scientific); mouse anti-chondroitin sulfate antibody (1:100 dilution, ab11570, Abcam); rabbit anti-TLR-2 polyclonal antibody (10 µg/ml, PA5-20020, Thermo Fisher Scientific), rabbit anti-TLR-4 monoclonal antibody (1:500 dilution, 76B357.1, Thermo Fisher Scientific), and rabbit anti-TLR-5 polyclonal antibody (10 µg/ml, 36-3900, Thermo Fisher Scientific).

Post incubation, membranes were washed with 1% BSA/PBS and incubated with 1:500 dilution of secondary antibodies (goat anti-mouse or goat anti-rabbit) conjugated with Alexa Fluor-488 (Thermo Fisher Scientific) at RT for 1.5 h. Membranes were washed then stained with Alexa Fluor-633 phalloidin (1:500 dilution, Thermo Fisher Scientific) at RT for 30 min to label filamentous actin followed by DAPI nucleic acid stain (DAPI dihydrochloride, 300 nM in PBS, Invitrogen, UK) at RT for 5 min. Filter membranes were washed then mounted with ProLong Glass antifade mountant (Invitrogen) and imaged on a Leica SP8 microscope. Images were acquired using 20x, 40x or 63x magnification with 0.3 µm Z step size giving 50% image overlap required for 3D image reconstruction. Images were processed using LAS X software version 3.5.7.

To analyse urothelial cell shedding, apical supernatants from a 12-well plate was collected and centrifuged at 300 g for 5 min. Pellets were re-suspended in 100 μl of PBS and cytocentrifuged onto a glass slide using a Shandon Cytospin 2 at 800 rpm for 5 min. Cells were fixed with 4% methanol-free formaldehyde for 15 min at RT then washed 3x in Hanks’ Balanced Salt solution (HBSS) (Thermo Fisher Scientific). Cells were stained with 5 µg/ml of Wheat Germ Agglutinin (WGA), Alexa Fluor-488 conjugate (Thermo Fisher Scientific) for 1 h at RT. Cells were washed with HBSS and stained with 2 µg/ml Hoechst (33342, Thermo Fisher Scientific) for 15 min at RT, and mounted as described above.

To aid in closer inspection of any aspect of the confocal reconstructions, all raw tiff data have been deposited into the open access University College London Data Repository (doi: 10.5522/04/22193692).

### 2.5 Transepithelial electrical resistance and paracellular permeability measurements

The TEER of the 3D-UHU model was measured using the EVOM3 with a STX2-Plus electrode (World Precision Instruments, UK) according to the manufacturer’s instructions. Briefly, a 1000 Ω test resistor was used to set up the equipment then blank handling mode was selected to allow subtraction of the blank control from the current resistance measurement of 3D cultures. Each measurement was recorded and stored on the device for further analysis.

Barrier integrity of 3D cultures was assessed using Fluorescein Isothiocyanate (FITC)-Dextran (MW 4,000, Sigma-Aldrich, UK). FITC-dextran solution (1 mg/ml) was prepared in urine and added to the apical chamber. Aliquots of medium from the basolateral side were collected in 0, 5, and 24 h post-infection (p.i.) (multiplicity of infection; MOI 10) and fluorescence was measured by a Tecan Spark microplate reader (Tecan, Switzerland) at an excitation of 485 nm and emission of 538 nm. The FITC-conjugated dextran concentration was presented as relative fluorescence unit (RFU).

### 2.6 Bacterial strains

To examine the barrier disruption and innate immune response to bacterial co-culture, several uropathogenic strains was employed. Uropathogenic *Escherichia coli* (UPEC) strains UTI89 (Mulvey et al., 2001), CFT073 (Mobley et al., 1990) (ATCC 700928), and *E. coli* 83972 (bei Resources) associated with asymptomatic bacteriuria (ABU) were grown in Luria-Bertani (LB, Sigma Aldrich) broth statically at 37°C for 48 h to induce expression of type 1 pili. Previously published clinical isolates were also tested: *Enterococcus faecalis* from a patient with UTI (*E. faecalis* 36 [EF36] (Lau et al., 2020); *E. faecalis* from an asymptomatic healthy male *E. faecalis* 77 [EF77] (Sathiananthamoorthy et al., 2019) and *Streptococcus agalactiae* from an asymptomatic healthy female (Sathiananthamoorthy et al., 2019) (Group B Streptococcus), were grown overnight in Tryptone Soya Broth (TSB, Thermo Fisher Scientific) at 37°C.

### 2.7 Co-culture and cytokine measurements

3D-UHU cultures were co-cultured with bacterial isolates at MOI 10. Initial seeding density was normally used to calculate MOI to aid in reproducibility; it should be noted, however, that the cells proliferate after this time, but equally the apical interface contains fewer cells (approximately 25,000-30,000 cells, as roughly calculated by surface area and cell size observed in microscopy images). To measure the expression of pro-inflammatory cytokines/chemokines, the apical supernatants were collected at 24 h post infection. Human Interleukin-8 (IL-8), IL-6, IL-1β, and TNF-α was measured by enzyme-linked immunosorbent assay (ELISA) per manufacture’s instruction (Biolegend, ELISA MAX Deluxe Set) and CXCL-1/GRO-α was measured using a R&D systems DueSet ELISA kit.

### 2.8 Statistical analysis

Data were analysed using GraphPad Prism 9. At least three independent biological replicates in technical replicates of two were performed for statistical analysis. Differences in TEER, FITC-dextran measurements and cytokine/chemokine expression between experimental samples and controls were analysed using 2way-ANOVA. Tukey’s multiple comparisons test was used to compare 3D-UHU TEER measurements in different passage numbers. Dunnett’s multiple comparisons test was used to compare each variable with the untreated control.

## 3 Results

### 3.1 An enhanced 3D-UHU microtissue model mimics human bladder epithelia

**Figure 1:**
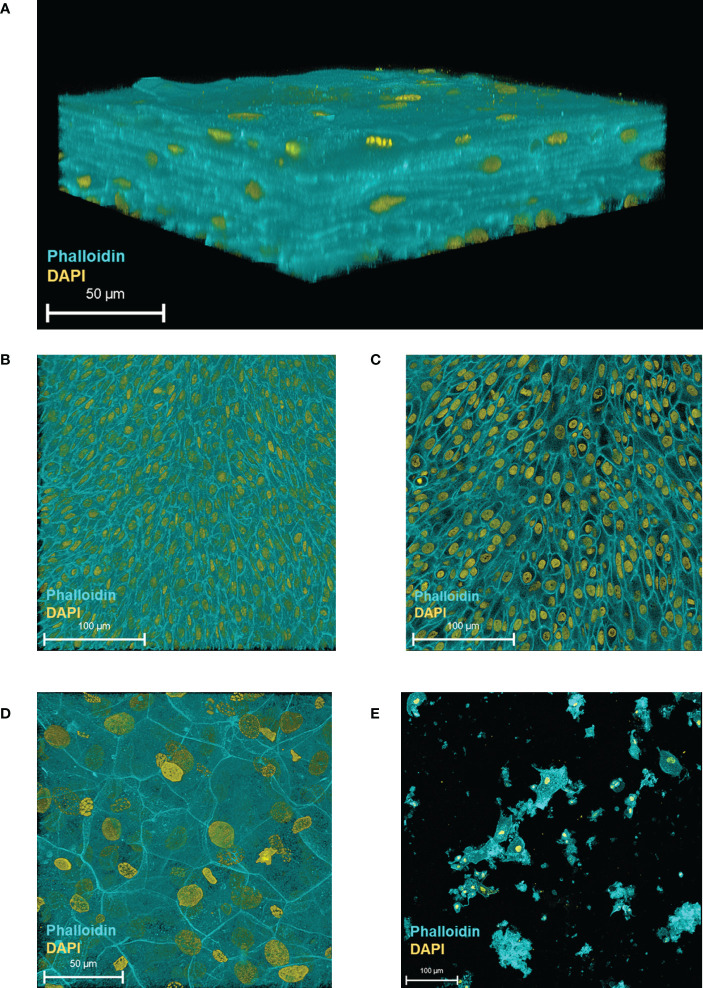
HBLAK cells differentiate, stratify and form a 3D urothelial microtissue model, 3D-UHU in 100% urine. **(A)** 3D confocal image of the urothelial model; **(B)** single optical slice at the lowest region of the model showing small, tightly packed basal cells; **(C)** single optical slice at the mid-section presenting slightly larger intermediate cells; **(D)** single optical slice at the apical surface exhibiting large, differentiated umbrella-like cells; **(E)** shed cells detected at day 18 in fully differentiated 3D models (supernatants collected from 12 models). Phalloidin-stained F-actin is presented in cyan and DAPI-stained DNA is presented in yellow; scale bars are as shown. Images are representative of at least three biologically independent experiments.

To assess 3D-UHU morphologically, we grew the 3D cultures for 18-20 days and then fixed and stained them for confocal microscopy. As shown in **Figure 1A**, we obtained stratified human bladder 3D models achieving up 7 cell layers thickness (**Figure 1A**). The single optical slices showed densely packed, very small basal cells in the basal layer (**Figure 1B**) followed by several layers (4-6) of slightly larger intermediate cells (**Figure 1C**), and large hexagonal umbrella-like cells forming the top layer (**Figure 1D**) (**Supplementary Movies 1, 2**). The movies revealed that there appeared to be a second layer of large cells directly below the apical layer that might also be umbrella-like cells, or intermediate cells on a continuum with umbrella cells, although uroplakin staining suggested only the most apical cells were terminally differentiated (see below). **Supplementary Figure 1** shows an example of how layer numbers were estimated. We imaged 3-6 randomly selected low-power (x20) microscopy fields derived from models created from four independent experiments, fixed and stained over more than a year’s worth of experiments, to assess whether the differentiated umbrella-like cell layer was, as our observations suggested, routinely homogeneous (**Supplementary Figure 2**). This assessment suggested that the umbrella-like cell layer was indeed homogeneous, with nearly 100% differentiation without the extensive zones of hypertrophy and undifferentiated monolayers seen in our previous version of this model (Horsley et al., 2018).

We examined epithelial cell shedding by collecting spent apical supernatants at regular intervals which were centrifuged onto glass slides prior to fixation and staining. A very low number of shed cells (~ 25 cells/well based on nuclei count, rest presumed cell debris) were detected on day 18 onwards (**Figure 1E**), suggesting that the model retains most of its apical cell layer (with shed cells representing approximately 0.1% of the predicted apical cell number).

In summary, the 3D-UHU model showed three distinct cell layers with discernible cell size differences and appropriate thickness, reminiscent of a human urothelium and remained largely intact throughout the culture process.

### 3.2 The 3D-UHU model expresses human bladder CD cell surface markers

**Figure 2:**
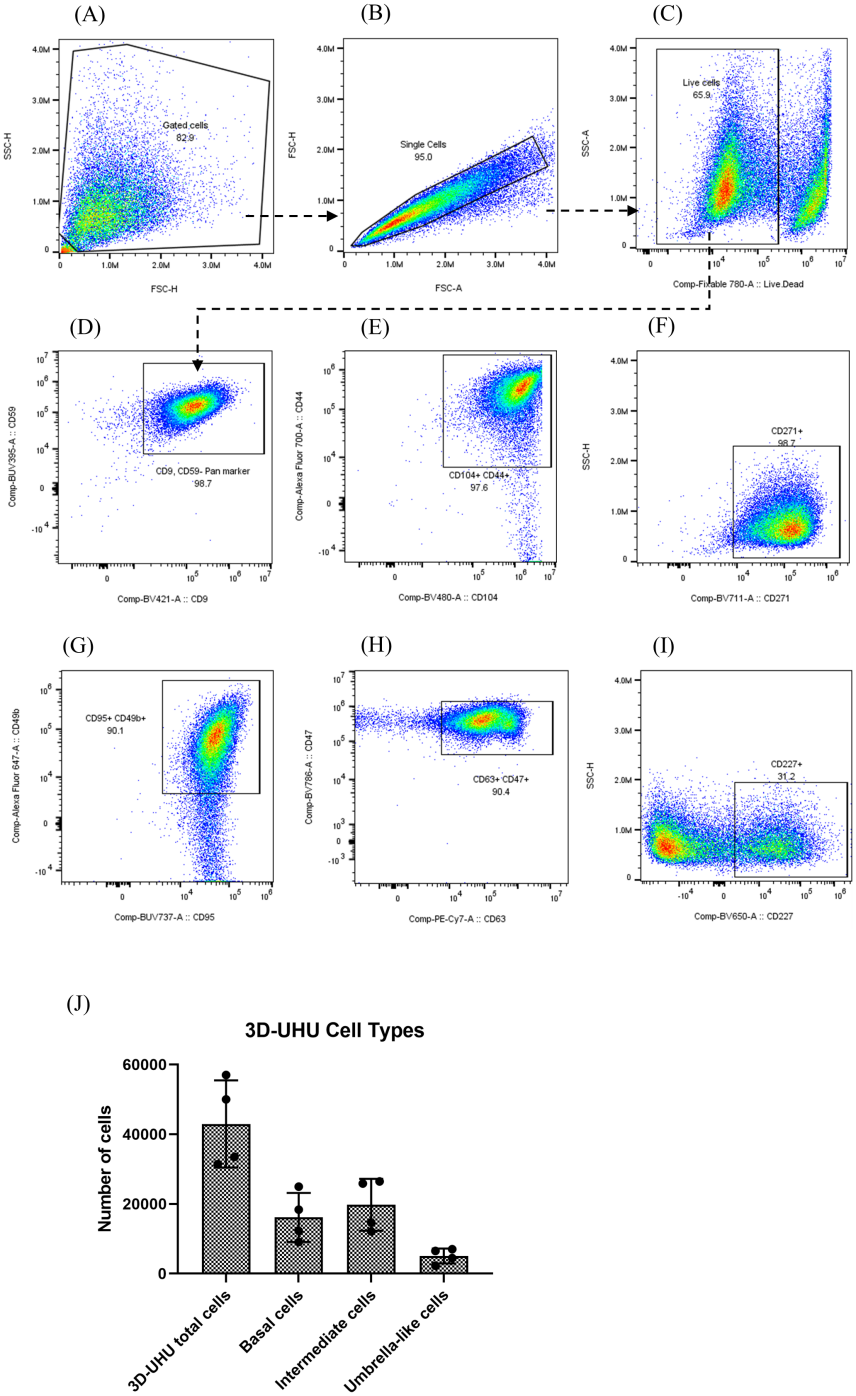
3D-UHU model expresses human bladder urothelial CD phenotypes. **(A–C)** gating strategy to profile urothelial CD markers by flow cytometry; **(D–I)** flow cytometric analysis of the expression of CD9/CD59 (pan cell surface markers); CD44/CD104, CD271 (basal cells); CD95/CD49b (intermediate cells); CD63/CD47, CD227 (umbrella cells); **(J)** number of basal cells (CD271^+^), intermediate cells (CD95^+^/CD49b^+^), and umbrella-like cells (CD227^+^) in 3D-UHU model; data represent mean ± SD, n=4 biologically independent experiments.

We identified stratified and terminally differentiated 3D-UHU cell types based on their marker expression using flow cytometric analysis. Dissociated single cells were first gated by size and granularity to exclude cellular debris. From the “gated cells”, singlets were sub-gated by their FSC-A/FSC-H properties. Single cells were plotted with the viability dye and cells considered “live” were selected (**Figures 2A–C**). Our choice of markers was informed by a paper from Liu and colleagues (Liu et al., 2012), who used a combination of transcriptomics and immunohistochemistry on human bladder tissue to comprehensively catalogue the cell surface markers expressed by different cell types in the human urothelium. From the “live” population, cells that were positive for CD9 and CD59 markers were selected (**Figure 2D**). CD9 and CD59 have been reported to be expressed by all three cell types in humans: basal, intermediate, and umbrella cells (Liu et al., 2012) (**Supplementary Table 2**). CD9, CD59 gated cells were plotted with CD44, CD104, and CD271 (**Figures 2E, F**). CD44 and CD104 have been described to be expressed strongly by basal cells in humans although intermediate cells similarly express these markers in low levels, whereas CD271 is expressed exclusively by basal cells (Liu et al., 2012) (**Supplementary Table 2**). The basal cells in 3D-UHU model were identified by expression of CD271 (**Figure 2F**). Intermediate cells have been reported to express a number of CD molecules in humans (e.g., CD9, CD46, CD49b, CD55, CD59, CD95, CD116, CD147) (Liu et al., 2012); however, these markers are also expressed by basal and umbrella cells to varying degrees of intensity (**Supplementary Tables 2A, B**). CD49b and CD95 markers (Liu et al., 2012) were used to label the intermediate cells (**Figure 2G**). To detect the umbrella-like cells, CD9/CD59 gated cells were plotted with CD47, CD63, and CD227 (**Figures 2H, I**). CD47 and CD63 markers have been shown to be expressed weakly by intermediate and basal cells in humans (**Supplementary Table 2B**). We identified the umbrella-like cells by CD227, the marker that is reported to be expressed exclusively by these cells in humans (Liu et al., 2012) (**Figure 2I**).

We used unstained, fluorescence minus one (FMO), and CD57 marker expressed by urothelial nerve sheath (data not shown) controls to set gate limits and rule out non-specific binding of the panel antibodies to the cellular surface. The number of each cell type was calculated from acquired flow cytometry data anlaysed by FlowJo (**Figure 2J**). Approximately 42,000 3D-UHU cells were acquired in total of which ~16,000 were CD271^+^ basal cells. Intermediate cells (CD95^+^/CD49b^+^) comprised the largest proportion of cell numbers (~20,000), which is consistent with there being multiple layers of this cell type, while a smaller number of cells (~5,000) comprised the CD227^+^ umbrella-like population, which is consistent with them forming one layer of very large cells. The median fluorescence intensity (MFI) of CD markers expressed by human bladder 3D model showed a high CD104 and a relatively low CD227 expression (**Supplementary Figure 3**).

### 3.3 The 3D-UHU model exhibits human urothelial biomarkers

**Figure 3:**
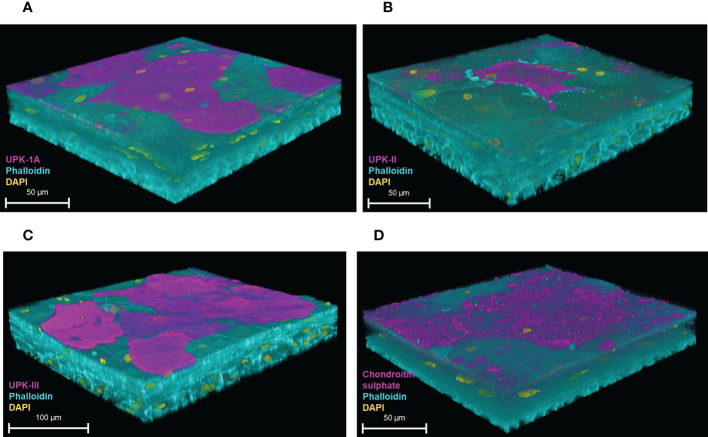
Uroplakin and GAG layer expression of the 3D-UHU model. 3D confocal image of **(A)** UPK-1A (magenta); **(B)** UPKII (magenta); **(C)** UPKIII (magenta); and **(D)** chondroitin sulphate (magenta) expressed at the apical surface of the 3D-UHU models. Phalloidin-stained F-actin is presented in cyan and DAPI-stained DNA is presented in yellow; scale bars are as shown. Images are representative of at least three biologically independent experiments.

We characterised the model further by examining the expression of urothelial-specific markers with confocal microscopy. The 3D-UHU model showed the expression of all three uroplakin proteins integral to formation of the AUM; uroplakin-1A (UPK1A) (**Figure 3A**), uroplakin-II (UPKII) (**Figure 3B**), and uroplakin-III (UPKIII) (**Figure 3C**) were expressed in a subset of umbrella-like cells with UPKIII exhibiting the highest surface expression followed by UPK-1A. Although studies have indicated that cultured urothelial cells do not express UPII (Hu et al., 2005), our model showed a modest expression of this marker. Next, we examined the GAG layer expression. The GAG layer consists of glycoproteins and glycolipids and is mainly composed of chondroitin/dermatan sulfate, heparan sulfate, keratan sulfate, and hyaluronic acid (Klingler, 2016). Although not all of these components were tested due to a lack of commercially available reagents, a chondroitin-rich GAG layer was detected at the umbrella-like cell layer (**Figure 3D**).

**Figure 4:**
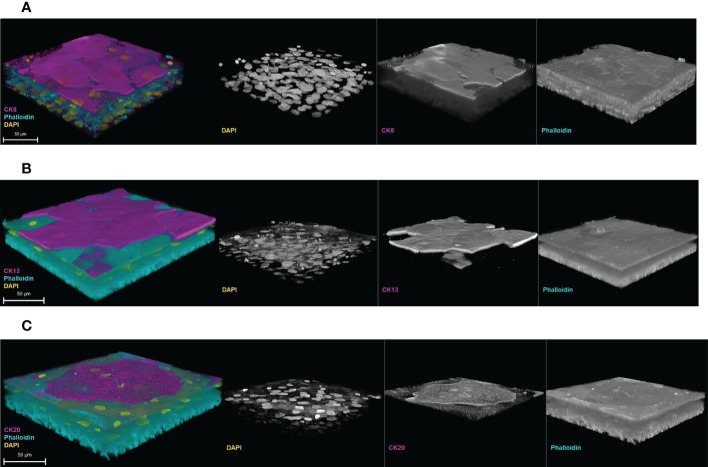
Cytodifferentiation in the 3D-UHU model. 3D confocal image of **(A)** CK8 (magenta) expressed throughout the layers; **(B)** CK13 (magenta) expressed at the late/terminal differentiated intermediate cell layers, and **(C)** CK20 (magenta) expressed at the terminally differentiated umbrella-like cells. Phalloidin-stained F-actin is presented in cyan and DAPI-stained DNA is presented in yellow; scale bars are as shown. Images are representative of at least three biologically independent experiments.

The 3D-UHU model also exhibited the correct spatial expression of cytokeratin (CK)8, 13, and 20 (**Figure 4**). CK8 was expressed throughout the basal, intermediate, and umbrella-like cell layers, though detected most strongly at the apical surface (**Figure 4A**) (**Supplementary Figure 4**). CK13, a differentiation marker indicating a switch from basal cells to intermediate cells which also has been reported to indicate cytodifferentiation of late intermediate cell layer (Varley et al., 2004; Varley and Southgate, 2008; Fishwick et al., 2017), appeared to be detected on the late differentiated intermediate cell layers (**Figure 4B**) (**Supplementary Figure 5**), not on the surface, while CK20, a terminal differentiation marker, was detected only at the apical surface layer, presented by umbrella-like cells (**Figure 4C**).

### 3.4 The 3D-UHU expresses adherens and tight junction proteins and forms a barrier

**Figure 5:**
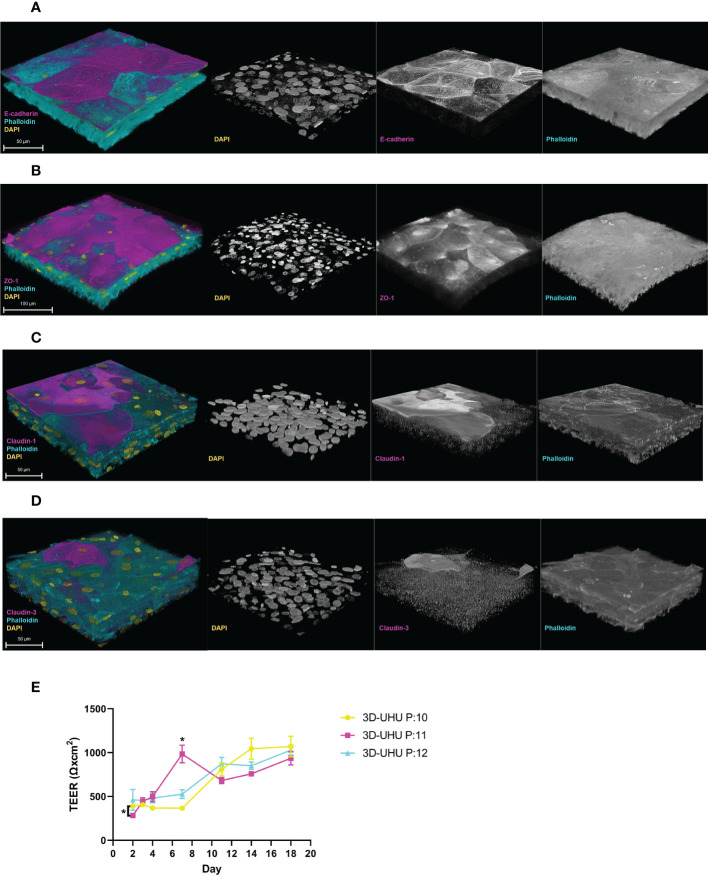
The 3D-UHU model expresses adherens and tight junction proteins and forms a barrier. **(A, B)** 3D confocal image of urothelial model expressing E-cadherin and ZO-1, respectively at the apical surface (magenta); **(C, D)** claudin 1 and claudin 3 presented throughout the layers (magenta). Phalloidin-stained F-actin is presented in cyan and DAPI-stained DNA is presented in yellow; scale bars are as shown. Images are representative of at least three biologically independent experiments. **(E)** TEER measurement of 3D-UHU model in a time-dependent manner on models from three different passages (P:10-12); ^*^ *p* < 0.05 calculated by two-way ANOVA followed by Tukey’s multiple comparisons test; data represent mean ± SD, n=3 biologically independent experiments.

The expression of adherens junction (AJ) E-cadherin and tight junction (TJ) proteins – ZO-1 and claudin 1, 3 – were examined by confocal microscopy (**Figures 5A–D**). In the literature, the umbrella cell layer is the only urothelial layer reported to form detectable tight and adherens junctions (Dalghi et al., 2020). The 3D-UHU model showed both membranous and cytoplasmic E-cadherin expression in the umbrella-like cells (**Figure 5A**). A strong ZO-1 expression was detected in cytoplasm of the umbrella-like cell layer with a faint staining at cell borders (**Figure 5B**), while claudin 1 and claudin 3 exhibited a discontinuous membrane and cytoplasmic staining and the expression was distributed diffusely throughout the strata (**Figures 5C, D**).

We determined the barrier integrity by measuring the transepithelial electrical resistance (TEER) in a time-dependent manner (**Figure 5E**). TEER was assessed on day 2 (48h post-seeding), day 3 (CnT-PR-3D addition to initiate cell differentiation), and days 4-18 (cell exposure to urine). Although the TEER time-course showed some passage differences around day 2 and 7 (*p* < 0.05), a stable increase in TEER was detected from day 12 to 18 reaching to ~1000 Ω.cm^2^ showing no significant inter-passage differences by the end.

### 3.5 The 3D-UHU expresses Toll-like receptors

**Figure 6:**
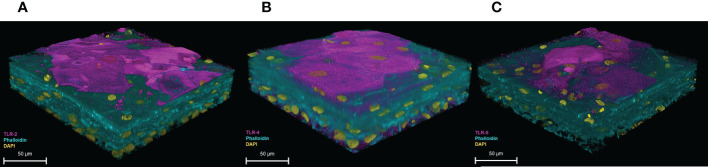
3D-UHU models express toll-like receptors. 3D-UHU expresses **(A)** TLR-2 (magenta); **(B)** TLR-4 (magenta); and **(C)** TLR-5 (magenta). Phalloidin-stained F-actin is presented in cyan and DAPI-stained DNA is presented in yellow; scale bars are as shown. Images are representative of at least three biologically independent experiments.

To examine the expression of TLRs, 3D-UHU models were stained for TLR-2, -4, and -5 (**Figure 6**). A moderate to strong expression of TLR-2 and TLR-4 was detected in 3D-UHU models (**Figures 6A, B**), while TLR-5 exhibited a low expression (**Figure 6C**).

### 3.6 Uropathogenic strains show variation in the disruption of the epithelial barrier

**Figure 7:**
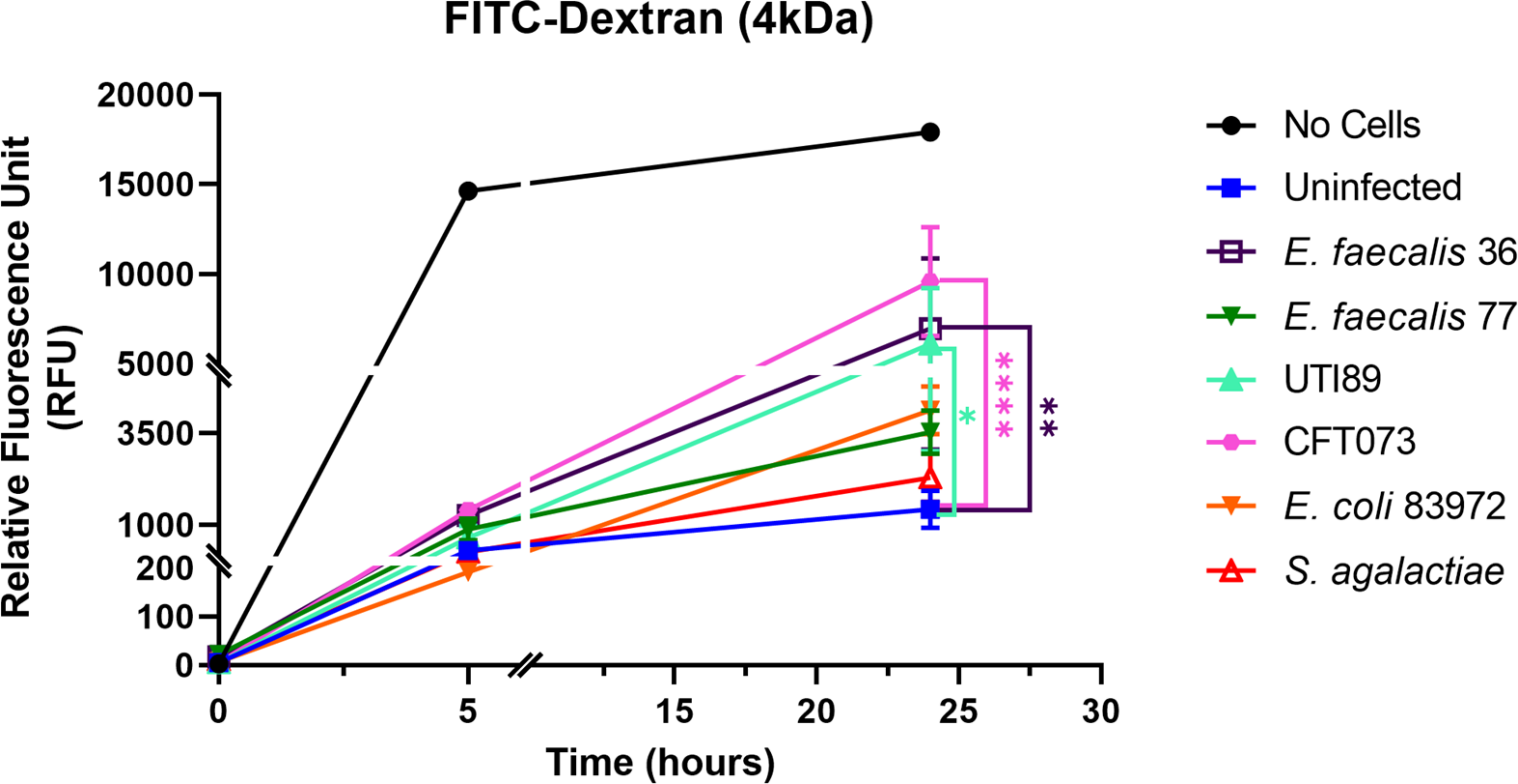
Uropathogens disrupt barrier integrity of 3D-UHU models. The barrier integrity in response to uropathogens (MOI 10) was measured by assessing the FITC-dextran influx into the basolateral chamber at 5h and 24h p.i. Data represent mean ± SD, n=3 biologically independent experiments. ^****^ *p* < 0.0001, ^**^ *p* < 0.01, *p < 0.05 calculated by two-way ANOVA followed by Dunnett’s multiple comparisons test.

Next, we wanted to evaluate the effect of bacterial infection on barrier integrity by assessing the paracellular permeability (**Figure 7**). A FITC-dextran (4 kDa) marker was used to measure tight junction permeability in response to uropathogenic *E. coli* CFT073 and UTI89, *E. faecalis* 36 and *E. faecalis* 77 clinical UTI isolates, *E. coli* 83972 (asymptomatic bacteriuria [ABU] isolate), and *S. agalactiae* isolated from urine of a healthy female (multiplicity of infection; MOI 10). At 5 h post-infection (p.i.), a similar but slight disruption in barrier integrity was detected with all strains compared with uninfected control cells. However, at 24 h p.i., the highly pathogenic CFT073 strain showed a significant (*p <*0.0001) disruption compared with the uninfected control, as did *E. faecalis* 36 (*p* < 0.01), and UTI89 (*p* < 0.05). *E. coli* 83972, *E. faecalis* 77, and *S. agalactiae* showed a barrier disruption that was not statistically significant compared with uninfected control.

### 3.7 Uropathogenic strains trigger an epithelial innate immune response

**Figure 8:**
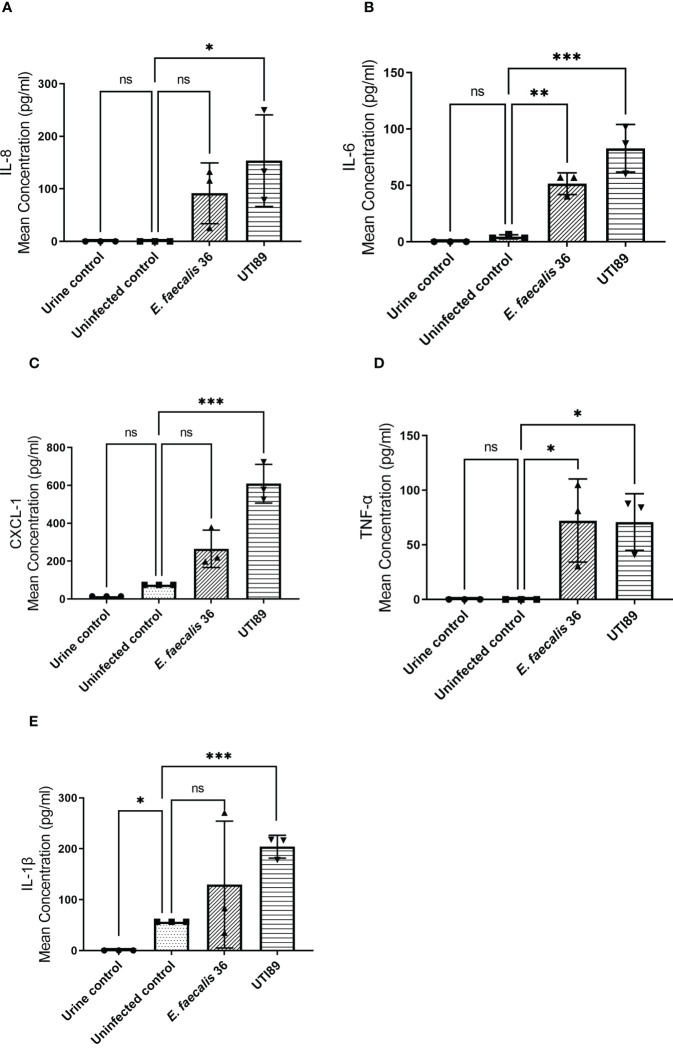
UTI89 and *E. faecalis* 36 trigger pro-inflammatory cytokine/chemokine responses. **(A)** IL-8; **(B)** IL-6; **(C)** CXCL-1; **(D)** TNF-α, and **(E)** IL-1β release was measured by ELISA in response to UTI89 and *E. faecalis* 36 strains (MOI 10) at 24h p.i. Data represent mean ± SD, n=3 biologically independent experiments. ^***^ *p* < 0.001, ^**^ *p* < 0.01, ^*^ *p* < 0.05, ns ≥ 0.05 calculated by two-way ANOVA followed by Dunnett’s multiple comparisons test.

To determine the 3D-UHU innate immune response to uropathogenic *E. coli* UTI89 and *E. faecalis* 36 (MOI 10), we measured proinflammatory IL-8, IL-6, CXCL-1, TNF-α, and IL-1β cytokine/chemokine levels in collected supernatants (**Figures 8A–E**). UTI89 caused a significant IL-8 (*p* < 0.05) (**Figure 8A**), IL-6 (*p* < 0.001) (**Figure 8B**), CXCL1 (*p* < 0.001) (**Figure 8C**), TNF-α (*p* < 0.05) (**Figure 8D**), and IL-1β (*p* < 0.001) (**Figure 8E**) response. Although *E. faecalis* 36 showed a significant IL-6 (*p* < 0.01) (**Figure 8B**) and TNF-α (*p* < 0.05) (**Figure 8D**) release, it caused an IL-8, CXCL-1, and IL-1β response (**Figures 8A, C, E**; respectively) that was not statistically significant compared with uninfected control.

## 4 Discussion

*In vitro* models of the human urothelium that physiologically resemble human tissue are becoming increasingly available. Such models generally have improved our understanding of human tissue development and have the potential to complement animal models (Knight and Przyborski, 2015). Several studies have taken advantage of cells lines and evaluated their capacity to form organoids. Smith et al. established a urothelial organoid using 5637 human bladder epithelial carcinoma cell line (HTB-9) under microgravity conditions (Smith et al., 2006), while Sharma et al. used this cell line to develop a human bladder-chip model (Sharma et al., 2021). Studies have also described the use of normal human urothelial (NHU) cells to develop a biomimetic urothelial tissue model (Cross et al., 2005) and human three-layered bladder assembloids (Kim et al., 2020). Here, we present a new advanced barrier-forming, urine-tolerant, 3D urothelial microtissue model. These experiments were performed after 18-20 days in urine culture, but longer time periods are possible if desired (we have used the model up to 4 weeks in our laboratory). This model showed morphological similarities with human bladder urothelium, stratifying to 7-8 layers with a single basal cell layer at the basal membrane, multiple intermediate cell layers and terminally differentiated umbrella-like cells at the apical surface. The model also displayed similar CD phenotypes to human bladder tissue (Liu et al., 2012), including CD271^+^ basal cells and CD227^+^ umbrella-like cells. Although the calculations were complicated by some markers also being expressed in other cell types, including challenges with distinguishing the intermediate cells, we estimated that the umbrella-like layer comprised about 11% of the total, whereas intermediate cells formed the majority (~ 47%), which is in line with expectations. Although we were able to characterise the cell populations using flow cytometric analysis and specific antibodies, comparing the flow cytometry panel to conventional histochemistry in future would help to distinguish the intermediate cells from other cell layers. One limitation of our model is that it is created from cells derived from just one donor; however, this can also be advantageous as it offers quality control and reproducibility that might not occur when using primary material from multiple donors. In principle, however, our protocol might be adaptable to primary material.

The prototype version of this model (Horsley et al., 2018) did not allow differentiation into a uniform umbrella-like cell layer; thus, as zones of monolayers occurred, the model could not maintain the barrier function needed to maintain 100% urine in the apical interface. After much trial and error, several relatively simple modifications enabled us to create thicker, homogeneous organoids in a reproducible manner. First, we found that growing the culture in 12-well Transwell format offered improvements over the original method. Second, we discovered that the cells possessed only a limited window, in terms of passage number, for being able to differentiate properly, specifically between passages 8 and 12 inclusive from thawing the commercial vial (translating to passage 33-37 considering that the vials are provided at passage 25). In the previous paper, cells were used up to passage 40-50 from factory vial thaw, typically much later than this window. Third, whereas the HBLAK cells in the previous paper were grown for only 14 days in urine, we achieved much better results culturing them for 18-20 days. Finally, in the previous study, cells normally took 3-5 days to become confluent before adding CnT-PR-3D medium, perhaps due to low seeding density or slow growth. We discovered, however, that any cultures that had not achieved confluency within 48 hours would perform poorly later, so we learned to routinely discard any slow-growing cultures.

Having achieved the improved model, we used immunofluorescence to assess a series of key biomarkers. In doing so, we noticed that the staining of a number of apical markers was discontinuous (i.e., seeming to be present on some cells and not on others). Although this could be the result of gaps left by recently shed cells, our analysis suggested exfoliation is very low in the absence of infection. Therefore the more likely explanation may be that, due to a surface that is not perfectly flat, the expression of these markers would have been detectable in a focal plane immediately below, which appears to be supported by the dynamic views offered by the image stacks (**Supplementary Movies 1, 2**). Alternatively, as it appeared that there might be another umbrella-like layer below the most apical layer, these could possibly comprise late intermediate cells in a continuum towards terminal differentiation. Very little is known, however, about how uniform these markers are in the human urothelium, nor how they might change as the cells ascend towards their final apical fate; future studies are warranted.

Uroplakins are the most prominent markers of urothelial differentiation (Wu et al., 2009). We detected the expression of three uroplakins (1A, II, and III) on the terminally differentiated umbrella-like cells in the 3D-UHU model. Remarkably, our model expressed UPKII, although studies exploring uroplakin expression have stated that in cultured urothelial cells, the glycosylation of pro-UPKII does not occur, thus hampering the formation of the uroplakin heterotetramer leading to impaired AUM assembly (Wu et al., 2009). It may be that the stratified and differentiated structure of this 3D model enables the correct environment for this glycosylation to occur as the process is differentiation-dependent (Wu et al., 2009). Furthermore, umbrella cells expressed a coating of chondroitin sulfate, a GAG layer marker found in human urothelium. Although some components of the GAG layer are difficult to stain by conventional antibodies, further studies into the detailed composition of this layer would be interesting.

Next, we examined the expression of cytokeratins, of which 20 isoforms are known to be expressed by human urothelium (Veranic and Jezernik, 2005). CKs are cytoskeletal polypeptides, and the specific pattern of their expression can be used to determine the cytodifferentiation of the urothelium (Varley and Southgate, 2008). Similar to human urothelium, our 3D model exhibited CK8, CK13, and CK20 markers. The 3D-UHU model showed CK8 expression throughout all layers while CK20 was exclusively expressed by terminally differentiated umbrella-like cells, as expected. The expression of CK13 has been described by a number of studies (Moll et al., 2008; Varley and Southgate, 2008; Fishwick et al., 2017); in the 3D-UHU model, a late differentiated transitional phenotype expressing CK13 seemed to be present, based upon the detection of expression at the late/terminal differentiated intermediate layer.

Adherens and tight junctions, together with desmosomes, function as a selective barrier in epithelial cells in controlling the paracellular diffusion of water, ions, and various macromoleculars (Buckley and Turner, 2018). Cadherins are located on the cell membrane and are important for the function of AJ. We examined the expression of E-cadherin in the 3D-UHU model and observed that it was distributed in the cytoplasm of the apical surface with a faint signal at cell perimeters. In addition, claudins are the main functional barrier-determining component of the TJ (Hustler et al., 2018) and claudin 3 expression has been shown to associate with differentiation and development of a tight barrier along with ZO-1 protein (Smith et al., 2015). The TJs, localised between the umbrella cells, contribute along with uroplakins to urothelial barrier function (Birder and Andersson, 2013). However, the intermediate cells, although expressing TJ-associated proteins such as claudins, do not form morphologically identifiable TJ or AJ, which is in contrast to the case in umbrella cells (Dalghi et al., 2020). We demonstrated that ZO-1, claudin 1 and claudin 3 expression in 3D-UHU model was localised to the apical surface together with diffuse expression throughout the cell layers, especially the claudins. Although a discontinuous and mainly cytoplasmic expression of the AJ and TJ markers were observed in the 3D-UHU model, examining the markers using sectioned membranes in future may provide an improved staining.

We further examined the TJ formation by TEER assessment. The 3D-UHU model showed a stable increase in TEER, achieving ~1000 Ω.cm^2^ around day 18-20. Urothelial models generated from three different subcultures (passage 10, 11 & 12) showed a similar trend and gained comparable TEER values on day 18. Although the 3D-UHU model does not reach the high TEER (usually > 2k Ω.cm^2^) that are reported in models using NHU cell cultures (Cross et al., 2005; Rubenwolf et al., 2012; Baker et al., 2014), unlike the NHU model, 3D-UHU is comprised of multiple layers, which might affect the overall TEER values. Further work is required to achieve a somewhat higher TEER value in the presence of full stratification; nevertheless, the moderate barrier function achieved was sufficient to exclude FITC-dextran well and to serve as a baseline for inspecting subsequent loss of barrier function after infection. One study revealed that the barrier integrity of rabbit urothelium mounted in an Ussing chamber was maintained even with a ten-fold drop in TEER as no significant leakage of biotin, fluorescein, or ruthenium was detected across the urothelium (Carattino et al., 2013). Indeed, when we examined the urothelial barrier function in response to both Gram-positive and Gram-negative uropathogens at MOI of 10, we found that CFT073 (a widely studied uropathogenic *E. coli* strain) (Mobley et al., 1990), *E. faecalis* 36 (a clinical isolate known to be invasive) (Horsley et al., 2018), and UTI89 (Mulvey et al., 2001) caused a significant barrier disruption at 24 h p.i.

Finally, we examined some aspects of urothelial innate immunity. First, we identified that 3D-UHU models express TLR-2, TLR-4, and TLR-5 although to varying levels, all of which are known to be important in urothelial defence in humans. In addition, we wanted to investigate the 3D-UHU innate immune response to infection challenge by two representative uropathogens, UTI89 and *E. faecalis* 36. UTI studies in human and mice have reported several cytokine/chemokine responses to urinary infection and their role in UTI including IL-6, IL-8, and IL-1 response during human UTI (Hedges and Svanborg, 1994; Abraham and Miao, 2015); the expression of IL-6 and IL-1α has also been detected in animal models of cystitis (Gonzalez et al., 2014). Here, a significant IL-8, IL-6, CXCL-1, TNF-α, and IL-1β release was detected in response to UTI89. With the *E. faecalis* 36 clinical isolate, infection induced statistically significant IL-6 and TNF-α levels similar to that secreted in response to UTI89. This cytokine/chemokine profile suggests that the 3D-UHU model mediates some human-relevant innate immune responses to uropathogenic stimuli, which may make it an appropriate *in vitro* model for understanding innate immune response at the mucosal-bacteria interface.

In conclusion, these data taken together suggest that our enhanced 3D-UHU microtissue model shares key structural and physiological features with human bladder urothelium and could be used as an *in vitro* model, alongside more traditional animal experiments and human clinical studies, to elucidate UTI host-pathogen interactions.

## Data availability statement

The original contributions presented in the study are included in the article/**Supplementary Material**. Further inquiries can be directed to the corresponding author.

## Author contributions

NJ designed and performed the experiments, analysed and interpreted the data, drafted the manuscript and figures. JR contributed to experimental design, discussion of concepts, initial drafting and final review of the manuscript and figures. All authors contributed to the article and approved the submitted version.
