# Supplementary material for: An immunoresponsive three-dimensional urine-tolerant human urothelial model to study urinary tract infection
Source: Front Cell Infect Microbiol. 2023 Mar 27;13:1128132. doi: 10.3389/fcimb.2023.1128132 (PMC10083561; doi:10.3389/fcimb.2023.1128132)
Supplement: Supplementary file 1 [file DataSheet_1.docx]

Supplementary Material

| **Antibodies** | **Optimised volume (µl) in 50** **µl of BD Horizon Brilliant Stain Buffer** |
| --- | --- |
| BV421 Mouse Anti-Human CD9 | 2.5 |
| Alexa Fluor 700 Mouse Anti-Human CD44 | 3 |
| BV786 Mouse Anti-Human CD47 | 5 |
| Alexa Fluor 647 Mouse Anti-Human CD49b | 3 |
| BUV395 Mouse Anti-Human CD59 | 2.5 |
| PE-Cy7 Mouse Anti-Human CD63 | 5 |
| BUV737 Mouse Anti-Human CD95 | 4 |
| BV480 Rat Anti-Human CD104 | 2.5 |
| BV650 Mouse Anti-Human MUC1 (CD227) | 10 |
| BV711 Mouse Anti-Human CD271 | 3 |

**Supplementary Table 1.** Selected antibodies to profile urothelial CD markers by flow cytometry and optimised volume per test.


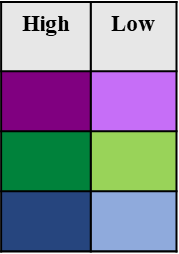

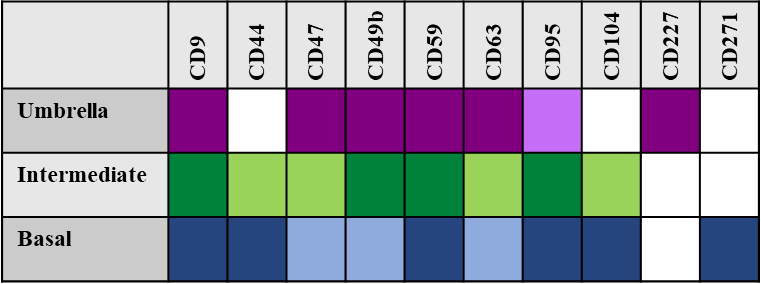

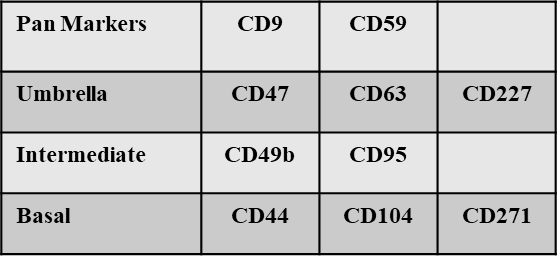


(A)

(B)

(C)

**Supplementary Table 2.** CD molecules expressed by different urothelial cell types. (A) Colours indicating high (bold) or low (light) expression of CD markers; (B) CD markers and level of expression in human bladder tissue (adapted from Liu *et al*., 2012); (C) selected CD markers to identify urothelial cell types in 3D-UHU model.


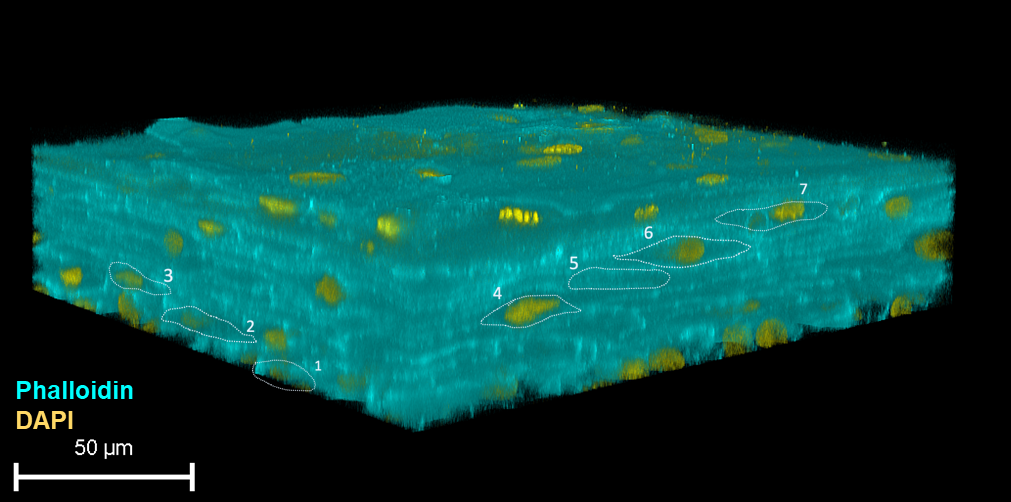
**Supplementary Figure 1.** HBLAK cells differentiate and stratify into 3D-UHU microtissue model with approximately 7 layers. Dotted outlines only indicate general presence in layer, not actual cell outlines. Estimation of each layer position was guided by horizontal membrane outlines alongside a nucleus either within the outline or at the same level laterally (as for 5). There appears to be an 8^th^ layer in this example, but as the interface is sometimes difficult to discern at this angle, we have not indicated it. See also the Supplementary movies for a more detailed view.


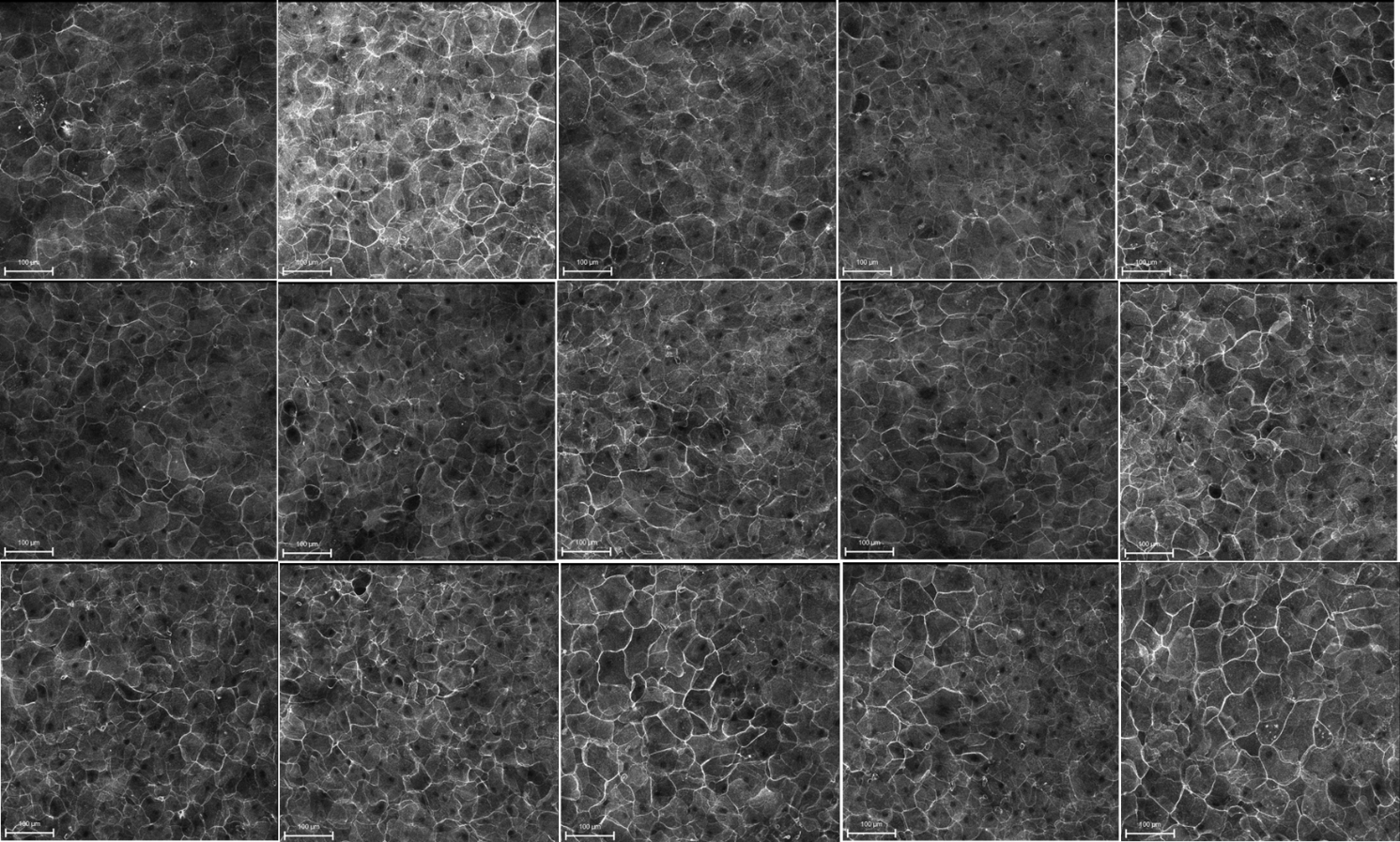


**Supplementary Figure 2.** HBLAK cells differentiate and stratify into the 3D-UHU microtissue model, which is homogenously differentiated at the apical layer. Single optical slices at the apical surface indicates differentiated umbrella-like cells without any areas of hyperplasia or undifferentiated monolayers. The images are representative of 3-6 different regions of four biologically independent experiments. Scale bar represents 100 µm.


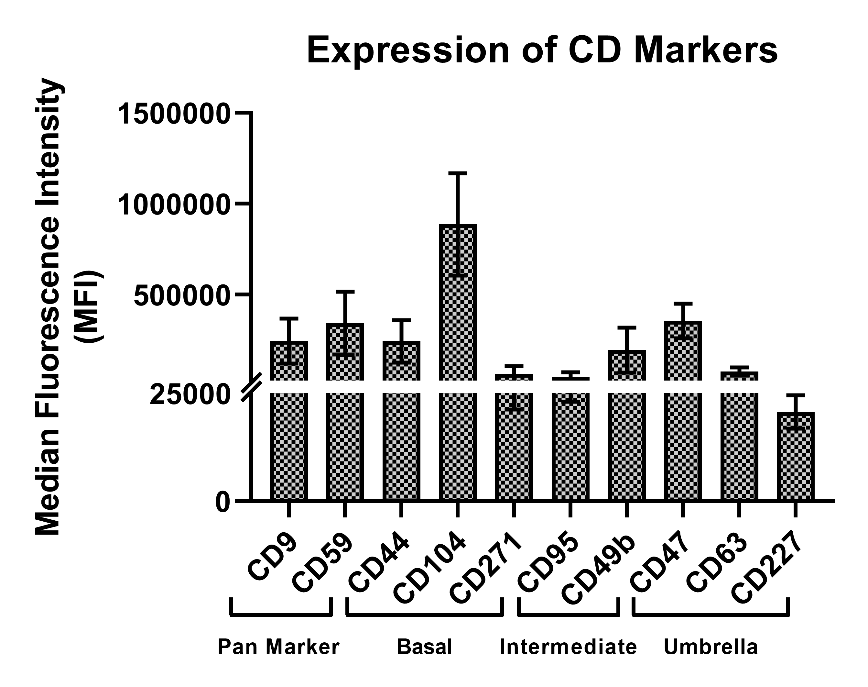


**Supplementary Figure 3.** Expression level (MFI) of CD cell surface markers; data represent mean ± SD, n=4 biologically independent experiments.

**
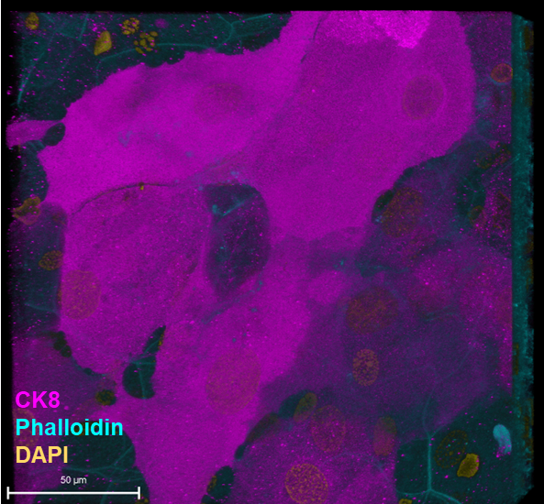

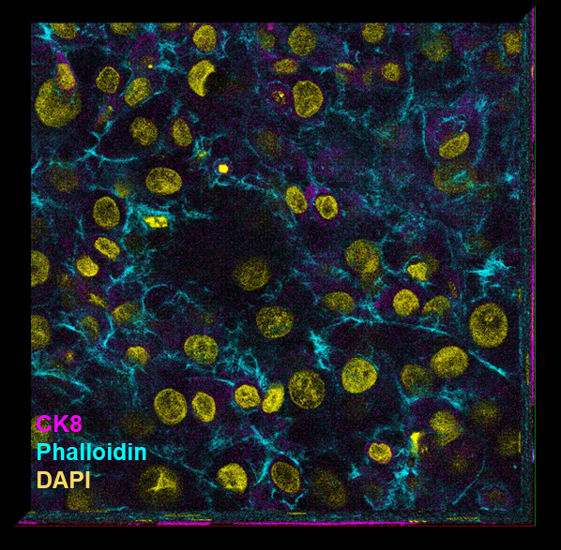

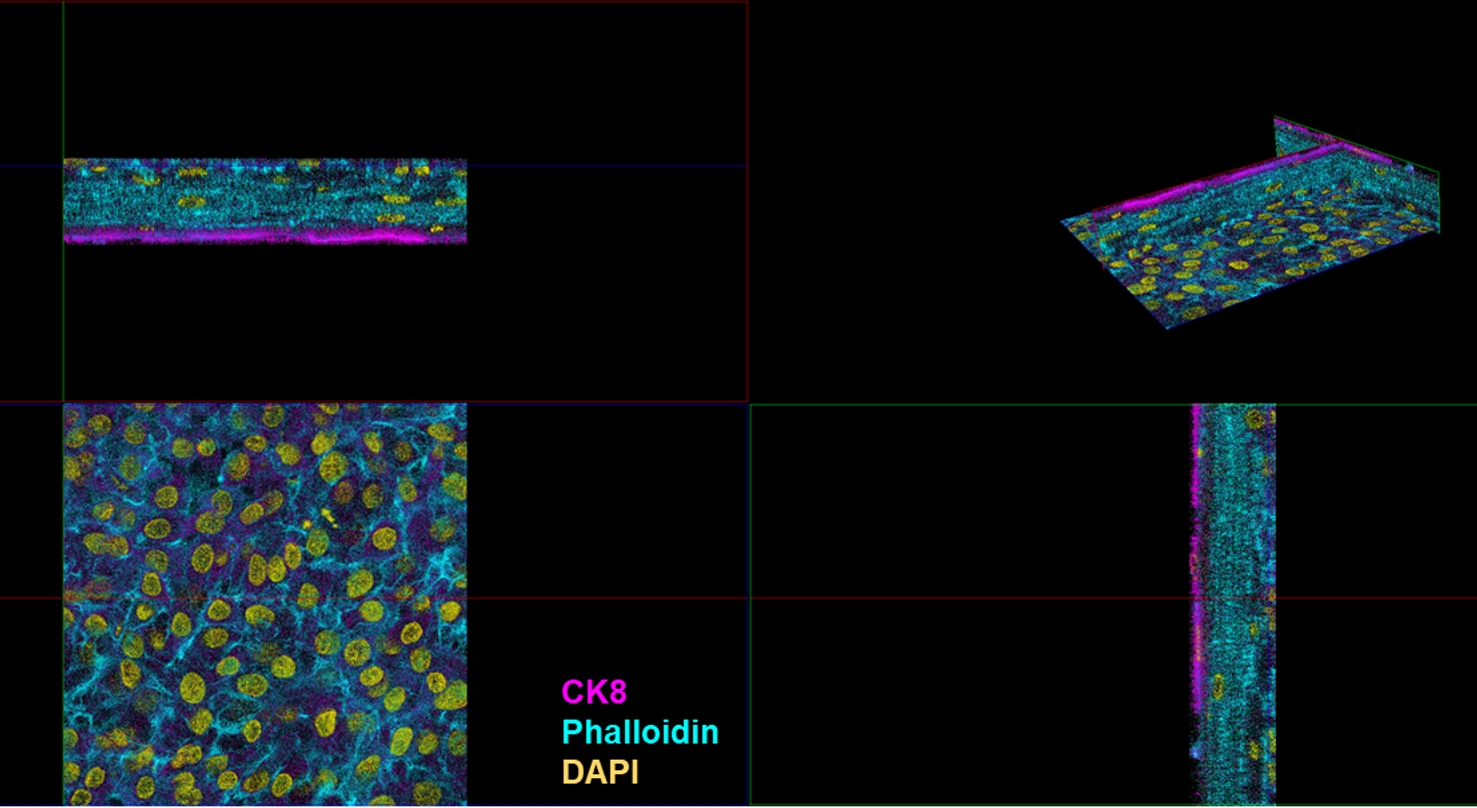
**

(F)

(E)

(D)

(C)

(B)

(A)

**Supplementary Figure 4.** CK8 expression in 3D-UHU model. (A) slice view (xz); (B) orthogonal cross sections of Fig. 4A; (C) single optical slice at the basal layer; (D) slice view (yz); (E) single optical slice at the apical region; (F) single optical slice at the mid-section. Orthogonal views exhibit expression of CK8 (magenta) throughout the layers.


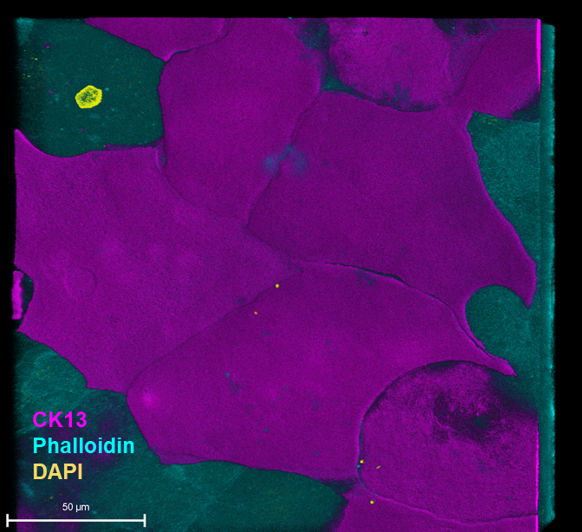

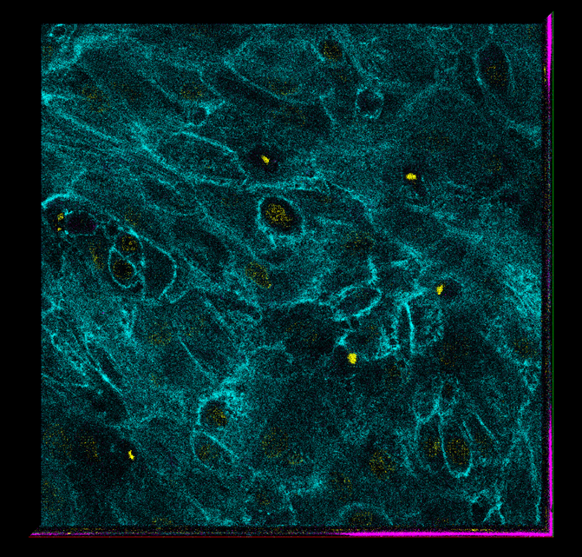
**
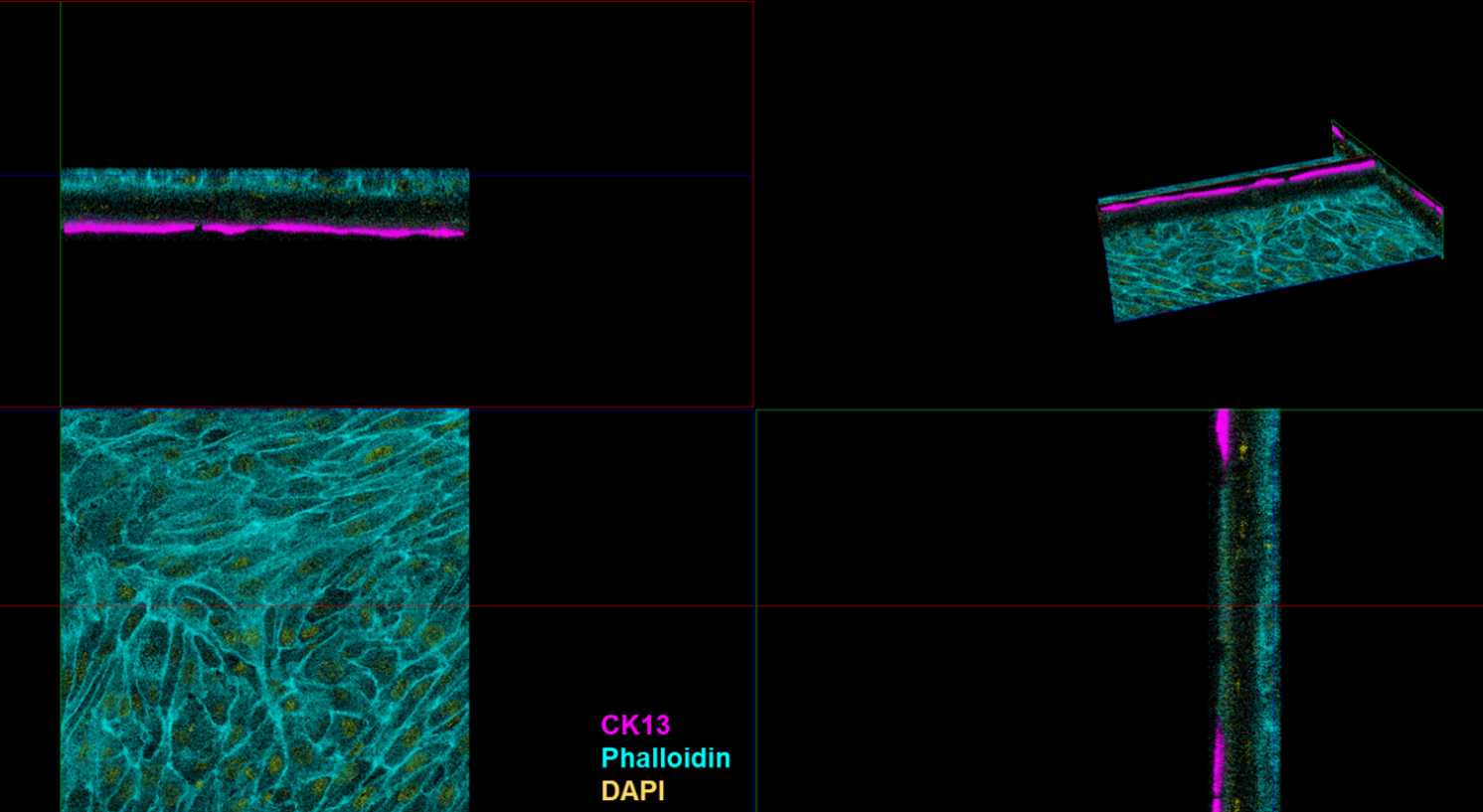
**

(F)

(E)

(D)

(C)

(B)

(A)

**Supplementary Figure 5.** CK13 expression in 3D-UHU model. (A) slice view (xz); (B) orthogonal cross sections of Fig. 4B; (C) single optical slice at the basal layer; (D) slice view (yz); (E) single optical slice at the apical region; (F) single optical slice at the mid-section. Orthogonal views (B) exhibit expression of CK13 (magenta) at the late/terminal differentiated intermediate layer.
